# Exploring the Efficacy of Large Language Models in Summarizing Mental Health Counseling Sessions: Benchmark Study

**DOI:** 10.2196/57306

**Published:** 2024-07-23

**Authors:** Prottay Kumar Adhikary, Aseem Srivastava, Shivani Kumar, Salam Michael Singh, Puneet Manuja, Jini K Gopinath, Vijay Krishnan, Swati Kedia Gupta, Koushik Sinha Deb, Tanmoy Chakraborty

**Affiliations:** 1 Department of Electrical Engineering Indian Institute of Technology Delhi New Delhi India; 2 Department of Computer Science & Engineering Indraprastha Institute of Information Technology Delhi New Delhi India; 3 YourDOST Karnataka India; 4 Department of Psychiatry All India Institute of Medical Sciences Rishikesh India; 5 Department of Psychiatry All India Institute of Medical Sciences New Delhi India; 6 Yardi School of Artificial Intelligence Indian Institute of Technology Delhi New Delhi India

**Keywords:** mental health, counseling summarization, large language models, digital health, artificial intelligence, AI

## Abstract

**Background:**

Comprehensive session summaries enable effective continuity in mental health counseling, facilitating informed therapy planning. However, manual summarization presents a significant challenge, diverting experts’ attention from the core counseling process. Leveraging advances in automatic summarization to streamline the summarization process addresses this issue because this enables mental health professionals to access concise summaries of lengthy therapy sessions, thereby increasing their efficiency. However, existing approaches often overlook the nuanced intricacies inherent in counseling interactions.

**Objective:**

This study evaluates the effectiveness of state-of-the-art large language models (LLMs) in selectively summarizing various components of therapy sessions through aspect-based summarization, aiming to benchmark their performance.

**Methods:**

We first created Mental Health Counseling-Component–Guided Dialogue Summaries, a benchmarking data set that consists of 191 counseling sessions with summaries focused on 3 distinct counseling components (also known as counseling aspects). Next, we assessed the capabilities of 11 state-of-the-art LLMs in addressing the task of counseling-component–guided summarization. The generated summaries were evaluated quantitatively using standard summarization metrics and verified qualitatively by mental health professionals.

**Results:**

Our findings demonstrated the superior performance of task-specific LLMs such as MentalLlama, Mistral, and MentalBART evaluated using standard quantitative metrics such as Recall-Oriented Understudy for Gisting Evaluation (ROUGE)-1, ROUGE-2, ROUGE-L, and Bidirectional Encoder Representations from Transformers Score across all aspects of the counseling components. Furthermore, expert evaluation revealed that Mistral superseded both MentalLlama and MentalBART across 6 parameters: affective attitude, burden, ethicality, coherence, opportunity costs, and perceived effectiveness. However, these models exhibit a common weakness in terms of room for improvement in the opportunity costs and perceived effectiveness metrics.

**Conclusions:**

While LLMs fine-tuned specifically on mental health domain data display better performance based on automatic evaluation scores, expert assessments indicate that these models are not yet reliable for clinical application. Further refinement and validation are necessary before their implementation in practice.

## Introduction

### Background

Counseling refers to a relationship between a professional counselor and individuals, families, or other groups that empowers the clients to achieve mental health, wellness, education, and career goals. Specifically, in individuals with psychological or interpersonal difficulties, mental health counseling may be seen as a key helping intervention. Counseling sessions embrace a client-centered approach, fostering an environment of trust and exploration. These sessions delve deep into personal experiences, where clients share intimate details while therapists navigate the dialogue to cultivate a safe and supportive space for healing. Discussions within these sessions span a wide range of topics, from recent life events to profound introspections, all of which contribute to the therapeutic journey. An important aspect of the counseling process lies in the documentation of counseling notes (summary of the entire session), which is essential for summarizing client stressors and therapy principles. Session notes are pivotal in tracking progress and in guiding future sessions. However, capturing the intricacies of these conversations poses a formidable challenge, demanding training, expertise, and experience of mental health professionals. These summaries distill key insights, including symptom and history (SH) exploration, patient discovery (PD), and reflection, while filtering out nonessential details. However, the need for meticulous recordkeeping can sometimes detract from the primary focus of therapy. Maintaining a seamless flow of conversation is paramount in effective therapy, where any disruption can impede progress. To streamline this process and ensure continuity, automation emerges as a promising solution for the counseling summarization task. While advances in artificial intelligence (AI) have revolutionized document summarization, the application of these technologies to mental health counseling remains relatively unexplored.

Previous studies [1-3] have recognized the potential of counseling summarization in optimizing therapeutic outcomes. However, existing models often overlook the unique nuances inherent in mental health interactions. Standard counseling dialogues, using reflective listening, involve identifying current issues; developing a biopsychosocial conceptualization, including past traumas and coping strategies; and chalking out treatment plans. The counseling dialogues also include discussion on between-session issues as well as crises, if any. An effective counseling summary should selectively capture information pertinent to each of these categories while eliminating extraneous details.

Despite the demonstrated capabilities of large language models (LLMs) in various domains, research in mental health counseling summarization is scarce. One major obstacle is the lack of specialized data sets tailored to counseling contexts. To bridge this gap, we embarked on a two-pronged approach: (1) creating a novel counseling-component–guided summarization data set, called Mental Health Counseling-Component–Guided Dialogue Summaries (MentalCLOUDS); and (2) evaluating state-of-the-art LLMs on the task of counseling-component–guided summarization. Through these efforts, we aim to propel the integration of AI technologies into mental health practice, ultimately enhancing the quality and accessibility of therapeutic interventions.

### Related Work

#### Overview

Summarizing counseling conversations enhances session continuity and facilitates the development of comprehensive therapy plans. However, analyzing these interactions manually is an arduous task. To address this challenge, advances in AI and natural language processing, particularly in summarization techniques, offer a promising solution. Summarization tasks can be approached via an extractive [4] or an abstractive [5] viewpoint. Extractive summarization involves identifying the most relevant sentences from an article and systematically organizing them. Given the simplicity of the approach, the resultant extractive summaries are often less fluent. By contrast, abstractive summarization extracts important aspects of a text and generates more coherent summaries. By using summarization, therapists can access recaps of sessions, sparing them the need to sift through lengthy dialogues. While summarization has been a long-studied problem in natural language processing [6], recent attention has shifted toward aspect-based summarization, a method that focuses on generating summaries pivoted on specific points of interest within documents.

Chen and Verma [1] proposed a retrieval-based medical document summarization approach in which the user query is fine-tuned using a medical ontology, but their method is limited due to its overall primitive design. Konovalov et al [7] highlight the importance of identifying emotional reactions and “early counseling” components. Strauss et al [8] used machine learning approaches to automate the analysis of clinical forms, and they envision using machine learning in mental health to a certain extent. Furthermore, research on major depressive disorder [9] underscores the significance of identifying crucial indicators from patient conversations, such as age, anxiety levels, and long episode duration, in the choice of the appropriate level of antidepressant medication, guiding subsequent sessions and prescriptions. Subsequently, the effectiveness of the prescribed antidepressants is monitored to assess the patient’s response.

This concept identifies crucial indicators from the patient’s conversations with the therapist and guides subsequent follow-up sessions based on the patient’s history of interactions and prescriptions. Deep learning approaches, such as the use of recurrent neural networks and long short-term memory, have been used to predict 13 predefined mental illnesses based on neuropsychiatric notes that contain 300 words each, on average, about the patient’s present illness and events associated with it, followed by a psychiatric review system that mentions the mental illness related to the patient [10]. Chen et al [11] proposed an extractive summarization approach using the Bidirectional Encoder Representations from Transformers (BERT) model [12] to reduce physicians’ efforts in analyzing tedious amounts of diagnosis reports. However, there remains a notable gap in effectively capturing medical information in session summaries.

In addition, some contemporary works used authentic mental health records to create synthetic data sets [13]. Afzal et al [14] reported the summarization of medical documents to identify PICO (Population, Intervention, Comparison, and Outcomes) elements. Manas et al [15] proposed an unsupervised abstractive summarization in which domain knowledge from the Patient Health Questionnaire-9 was used to build knowledge graphs to filter relevant utterances. A 2-step summarization was devised by Zhang et al [16] wherein partial summaries were initially consolidated, and the final summary was generated by fusing these chunks. Furthermore, Zafari and Zulkernine [17] demonstrated a web-based application built using information extraction and annotation tailored to the medical domain.

For dialogue summarization, abstractive summarization has been the de facto standard due to its ability to capture critical points coherently. Nallapati et al [18] used an encoder-decoder–based abstractive summarization method, which was further improved via the attention mechanism [19]. Subsequently, See et al [20] introduced a hybrid approach of extractive and abstractive summarization. Chen and Bansal [2] proposed a reinforcement learning-based approach as a mixture of extractive and abstractive approaches for summarization wherein emphasis is given to redundancy reduction in the utterances extracted from the conversation. Recent research reveals the dependence of specific utterances in the extraction of salient sentences from the conversation utterances. In this regard, Narayan et al [3] analyzed topic distribution based on latent Dirichlet allocation [21]. Subsequently, Song et al [22] segregated utterances into 3 labels: problem description, diagnosis, and other. In medical counseling, Quiroz et al [23] and Krishna et al [24] adopted the method of selecting significant utterances for summarizing medical conversations.

In aspect-based summarization, instead of an overall summary of the entire document, summaries at different aspect levels are made based on specific points of interest. These aspects could be movie reviews [25-28] or summarization guided by different domains [29,30] where the documents or the segments of the documents are tagged with these aspects. Hayashi et al [31] released a benchmarking data set on multidomain aspect-based summarization where they annotated 20 different domains as aspects using the section titles and boundaries of each article chosen from Wikipedia. Frermann et al [29] reported an aspect-based summarization of the news domain. Their method can segment documents by aspect, and the model can generalize from the synthetic data to natural documents. The study further revealed the models’ efficacy in summarizing long documents. Recently, aspect-based summarization has garnered considerable traction; however, the data set is limited. Yang et al [32] released a large-scale, high-quality data set on aspect-based summarization from Wikipedia. The data set contains approximately 3.7 million instances covering approximately 1 million aspects sourced from 2 million Wikipedia pages. Apart from releasing the data set, the authors also benchmarked it on the Longformer-Encoder-Decoder [33] model where they performed zero-shot, few-shot, and fine-tuning on 7 downstream domains where data are scarce. Joshi et al [34] address the general summarization of medical dialogues. They proposed combining extractive and abstractive methods that leverage the independent and distinctive local structures formed during a patient’s medical history compilation. Liu et al [35] reported a topic-based summarization of general medical domains pertaining to topics such as swelling, headache, chest pain, and dizziness. Their encoder-decoder model tries to generate 1 symptom (topic) at a time. Besides, work on formalizing the conversation text has been reported in the study by Kazi and Kahanda [36]. This work treats the formalization of the case notes from digital transcripts of physician-patient conversations as a summarization task. The method involves 2 steps: prediction of the electronic health record categories and formal text generation. Gundogdu et al [37] used a BERT-based sequence-to-sequence model for summarizing clinical radiology reports. The experimental results indicated that at least 76% of their summary generations were as accurate as those generated by radiologists. There is also a report on topic-guided dialogue summarization for clinical physician-patient conversations [38]. The approach first learns the topic structure of the dialogues and uses these topics to generate the summaries in the desired format (eg, the subjective, objective, assessment, and plan format). Zhang et al [39] proposed a method for factually consistent summarization of clinical dialogues. This method involves extracting factual statements and encoding them into the dialogue. In addition, a dialogue segmenter is trained to segment the dialogues based on topic switching, which enhances the model’s overall discourse awareness. Chintagunta et al [40] used GPT-3 [41] to generate training examples for medical dialogue summarization tasks. Recently, there have been reports of LLMs being used in medical dialogue summarization to expedite diagnosis by focusing on relevant medical facts, thereby reducing screening time [42]. The authors conducted benchmarking on GPT-3.5, Bidirectional and Auto-Regressive Transformer (BART) [43], and BERT for Summarization [44]. The study indicated that GPT-3.5 generated more accurate and human-aligned responses than the other 2 models. Another study [45] demonstrated the effectiveness of LLMs in clinical text summarization across 4 different tasks: physician-patient dialogue, radiology reports, patient questions, and progress notes. The quantitative analysis revealed that the summaries generated by the adapted LLMs were comparable, or even superior, in quality to those of the human experts in terms of conciseness, correctness, and completeness. Singh et al [46] used open-source LLMs to extract and summarize suicide ideation indicators from social media texts to expedite mental health interventions.

#### Opportunities

The aforementioned previous works either did not focus on aspect-based summarization or reported on general clinical discussions of common symptoms and conditions (eg, cough, cold, and fever). However, there are still avenues to be explored in the aspect-based summarization of mental health therapy conversations, considering that mental health is a pressing global issue requiring urgent consideration. These therapy conversations encompass several counseling components, including patient information, past symptoms, diagnosis history, reflection, and the therapist’s action plans. Focusing the summaries on these counseling components would facilitate targeted and focused summaries, significantly reducing the time and effort and leading to more effective therapy overall. In this direction, our work is motivated by the study conducted by Srivastava et al [47], which reported on a summarization-based counseling technique from therapist-client conversations. They released a conversation data set that is structured with the core components of psychotherapy about SH identification or the discovery of the patient’s behavior. The authors proposed an encoder-decoder model based on Text-to-Text Transfer Transformer (T5) [48] for their counseling-component–guided summarization model. However, a single, generic summary is generated in the work, and no focus is given to generating aspect-based summaries. Consequently, we extended the work by using the counseling components, namely SH exploration, PD, and reflection, into an aspect-based summarization framework. To this end, we created MentalCLOUDS, a data set that incorporates summaries aligned with the distinct counseling components. We also explored the efficacy of the state-of-the-art LLMs (encoder-decoder as well as decoder-only models) for the summarization of counseling dialogues in this work.

#### Taxonomy

**Figure 1:**
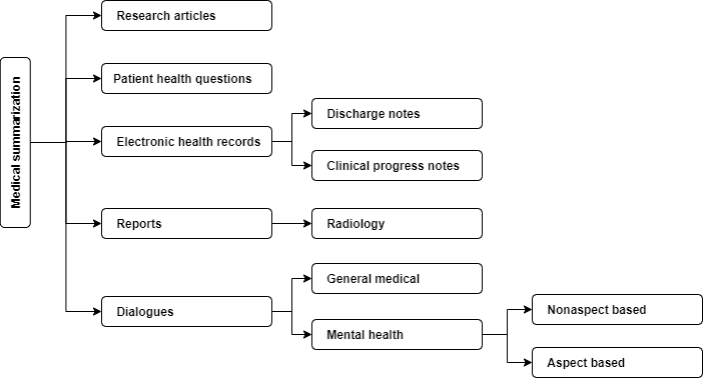
Taxonomy of summarization methods in the medical domain.

On the basis of the survey of related works on summarization in the medical domain in general and in mental health in particular, we present a taxonomy of task formulations for summarization tasks in the medical domain (Figure 1 [11,15,22-24,34,37,39,40,45,47,49-68]). In general, medical text summarization is divided into research articles [49-52], reports, patient health questions, electronic health records, and dialogue summarization. Report summarization encompasses the summarization of reports, such as impressions or summarizations of radiology findings [37,45,53-55]. Patient health question summarization involves summarizing informal, nontechnical, and lengthy patient questions into technically sound and concise ones [56-59]. Electronic health record summarization includes the summarization of patient notes such as clinical progress notes [60-63] and discharge notes [11,53,64-66]. Our work focuses on the abstractive dialogue summarization of mental health counseling conversations, specifically targeting the counseling aspects. In addition, the survey includes general medical dialogue summarization [22-24,34,39,40,45] and mental health dialogue summarization [15,47,67,68]. Of note, this taxonomy does not represent the global scenario but rather provides a comprehensive depiction based on the aforementioned survey.

#### Challenges

Mental health counseling conversations often involve sensitive and confidential information. There is an expectation of empathetic and reflective responses from the therapist and action plans based on which the therapy is conducted. Generative AI–based counselors are susceptible to generating insensitive or incorrect suggestions and lacking empathy in their responses, which can negatively impact the therapy process. Moreover, the components or aspects of counseling sessions are subjective, and a counseling conversation can have multiple aspects. Therefore, the scope of the aspect-based summarization is limited to the specific annotated aspects. However, annotating these aspects requires expert manual intervention, which is costly both in terms of human resources and the financial perspective.

## Methods

### Overview of the Proposed Data Set: MentalCLOUDS

To evaluate the performance of diverse summarization systems across various aspects of counseling interactions, we expanded upon the Mental Health Summarization (MEMO) data set [47]. Comprising 11,543 utterances extracted from 191 counseling sessions involving therapists and patients, this data set draws from publicly accessible platforms such as YouTube. Embracing a heterogeneous demographic spectrum with distinctive mental health concerns and diverse therapists, the data set facilitates the formulation of a comprehensive and inclusive approach for researchers. Using preprocessed transcriptions derived from counseling videos, the constituent dialogues within the data set exhibit a dyadic structure, exclusively featuring patients and therapists as interlocutors. Within each conversation, 3 pivotal counseling components (aspects) emerge: SH exploration, PD, and reflective utterances.

**Figure 2:**
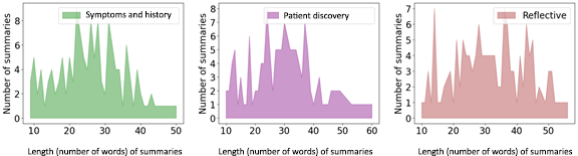
Distribution of summary lengths in the Mental Health Counseling-Component–Guided Dialogue Summaries (MentalCLOUDS) data set.

Our study aims to capture the essence of each aforementioned counseling component, embarking on the creation of 3 distinct summaries for a single dialogue, with each summary tailored to a specific counseling component. Expanding upon the MEMO data set, we augmented it with annotated dialogue summaries corresponding to the 3 identified components. Collaborating closely with a team of leading mental health experts (for their details, refer to the Qualitative Assessment by Experts subsection), we crafted annotation guidelines and subjected the summary annotations to rigorous validation processes. We call the resultant data set MentalCLOUDS. We highlight its key statistics in Table 1 and Figure 2.

**Table 1:** Statistics of the Mental Health Counseling-Component–Guided Dialogue Summaries data set.

Set	Dialogues (n=191), n (%)	Utterances (n=11,543), n (%)	Utterances per dialogue, mean (SD)	Patient utterances (n=5722), n (%)	Therapist utterances (n=5814), n (%)	SH^a^ utterances (n=2379), n (%)	PD^b^ utterances (5428), n (%)	Reflective utterances (n=1242), n (%)
Training	131 (68.59)	8342 (72.3)	63.68 (38.44)	4124 (72.1)	4211 (72.4)	1882 (79.1)	3826 (70.5)	884 (71.2)
Validation	21 (10.99)	1191 (10.3)	56.71 (27.06)	594 (10.4)	597 (10.3)	206 (8.7)	445 (8.2)	146 (11.8)
Test	39 (20.42)	2010 (17.4)	51.53 (39.96)	1004 (17.5)	1006 (17.3)	291 (12.2)	1157 (21.3)	212 (17.1)

^a^SH: symptom and history.

^b^PD: patient discovery.

### Data Annotation Process

#### Guidelines

Conversations in counseling situations can be challenging, given the sensitive nature of the information shared. A therapist’s reflective and open attitude can facilitate this expression. This dynamic is reinforced by the proposed MentalCLOUDS data set. This data set distinguishes the utterances dedicated to symptom exploration, discovering the history of mental health issues and patient behavior, as well as providing insights into past narratives, thereby shaping the patient’s present circumstances. These nuanced elements form the core of our identified counseling components. To improve the richness of the data set, we collaborated with mental health experts to formulate a set of annotation guidelines [69]. Furthermore, these guidelines serve as a comprehensive framework by which annotators can focus their attention on particular aspects of the conversation that are essential for producing summaries that are customized for each counseling component. By adhering to these guidelines, the therapeutic techniques are captured in the annotations. This ensures that the resulting summaries are concise yet rich in informative content for the specific component.

#### Psychotherapy Elements

Within the realm of mental health therapy sessions, distinct counseling components play a pivotal role in facilitating successful interventions. The MentalCLOUDS data set serves as a valuable resource, furnishing meticulously labeled utterances that encompass 3 fine-grained components [47]:

- SH: this facet encapsulates utterances teeming with insightful information crucial for the therapist’s nuanced assessment of the patient’s situation.
- PD: patients entering counseling sessions often bring intricate thoughts to the fore. Therapists, in turn, endeavor to establish therapeutic connections, creating a conducive environment for patients to articulate and unravel their thoughts. Such utterances by the therapist that encourage patients to reveal their concerns lie in this category.
- Reflecting: therapists use concise utterances, allowing ample space for patients to share their life stories and events. Encouraging patient narratives, therapists may also use hypothetical scenarios to evaluate actions and enhance understanding.

When crafting a summary for a dialogue *D*, aligned with a specific counseling component *C*, our primary focus rests on utterances marked with *C* within *D* in the MEMO data set. Consequently, we derived 3 distinct counseling summaries for each counseling component within a single session to create the MentalCLOUDS data set. Table 1 shows the data statistics, where a balanced distribution of patient and therapist utterances within the data set is evident. Notably, PD emerges as the prevailing label in the data set, highlighting patients’ inclination to discuss ancillary topics rather than focusing solely on their mental health concerns when prompted to share their experiences. By contrast, reflecting emerges as the least tagged label in this comprehensive analysis.

### Benchmarking

In recent years, the spotlight on LLMs has intensified, captivated by their extraordinary performance across diverse applications. From classification tasks such as emotion recognition [70] to generative problems such as response generation [71], these models have proven their versatility. In this paper, our focus is directed toward evaluating their capability in the domain of counseling summarization, specifically using MentalCLOUDS. In our comprehensive analysis, we leveraged 11 state-of-the-art pretrained LLM architectures, including a mix of general-purpose and specialized models. These models are considered to carefully assess their performance concerning each facet of the counseling-component summaries. We explain each of these systems in Textbox 1.

This is to highlight that all baseline models are transformer based, and computational complexities associated with the transformer-based architectures while being trained or fine-tuned involve a computational cost of *O*(*L* × *N*^2^ × *D*), where *N* represents the sequence length, *D* denotes the hidden dimension, and *L* signifies the number of transform layers. As we maintain a constant number of layers across all training steps, the computational complexity simplifies to *O*(*N*^2^ × *D*).

Moreover, our selection of benchmarked models comprises both small language models (SLMs), such as BART, T5, the GPT family, Phi-2, and MentalBART, as well as LLMs such as Flan-T5, Mistral, Llama-2, and MentalLlama. SLMs typically operate within the parameter range of 300 million to 2 billion, whereas LLMs are characterized by a higher parameter count, ranging from 7 billion to 9 billion (as kept in our study). In addition to analyzing the models’ complexity for a better understanding of their applicability, another crucial metric to consider is the model’s runtime. LLMs tend to consume more runtime due to their larger parameter count, while SLMs run quickly but may compromise accuracy. A comprehensive analysis of the models’ runtime is provided in Table 2.

**Description of the 11 models evaluated.**

- Bidirectional and Auto-Regressive Transformer (BART) [43]: this is a sequence-to-sequence model designed for various natural language processing (NLP) tasks, including text summarization. It uses a transformer architecture with an encoder-decoder structure. It incorporates a denoising autoencoder objective during pretraining, reconstructing the original input from corrupted versions. We used the pretrained base version of the model in our experiments.
- Text-To-Text Transfer Transformer (T5) [48]: this is a versatile transformer-based model consisting of an encoder-decoder framework with bidirectional transformers. It reframes all NLP tasks as text-to-text tasks, providing a unified approach. T5 learns representations by denoising corrupted input-output pairs. Its encoder captures contextual information while the decoder generates target sequences. The pretrained base version of T5 was used in our experiments.
- GPT-2 [72]: this is a transformer-based language model that comprises a stack of identical layers, each with a multihead self-attention mechanism and position-wise fully connected feed-forward networks. GPT-2 follows an autoregressive training approach, predicting the next token in a sequence given its context.
- GPT-Neo [73]: trained from the Pile data set [74], GPT-Neo exhibits a similar architecture as GPT-2 except for a few modifications, such as the use of local attention in every other layer with a window size of 256 tokens. In addition, GPT-Neo houses a combination of linear attention [75], a mixture of experts [76], and axial positional embedding [77] to achieve performance comparable to that of larger LLMs, such as GPT-3.
- GPT-J [78]: this is a transformer model trained using the methodology proposed by Wang [78]. It is a GPT-2–like causal language model trained on the Pile data set.
- FLAN-T5 [79]: this is the instruction fine-tuned version of the T5 model with a particular focus on scaling the number of tasks, scaling the model size, and fine-tuning on chain-of-thought data.
- Mistral [80]: this is a decoder-based LLM with a sliding-window attention mechanism, where it is trained with an 8k context length and fixed cache size, with a theoretical attention span of 128K tokens. Faster inference and lower cache are ensured by using grouped query attention [81].
- MentalBART [82]: this is an open-source LLM constructed for interpretable mental health analysis with instruction-following capability. The model is fine-tuned using the Interpretable Mental Health Instruction (IMHI) data set [82] and is expected to make complex mental health analyses for various mental health conditions.
- MentalLlama [82]: similar to MentalBART, MentalLlama is the counterpart of the Llama architecture but is trained on the IMHI data set. The model is fine-tuned to integrate the capability of an LLM with domain knowledge in mental health.
- Llama-2 [83]: this is an auto-regressive language model that uses an optimized transformer architecture. The tuned versions use supervised fine tuning [84] and reinforcement learning with human feedback [85] to align with human preferences for helpfulness and safety. The model is trained exclusively on publicly available data sets.
- Phi-2: this is an extension of Phi-1 [86]. Phi-1 is a transformer-based frugal LLM with the largest variant having 1.3 billion parameters. It is trained on textbook-quality data. It emphasizes the quality of the data to compensate for its relatively small number of parameters. Phi-2 has 2.7 billion parameters, which shows comparable performances with other larger LLMs despite its smaller size.

**Table 2:** Average runtime of models fine-tuned on Mental Health Counseling-Component–Guided Dialogue Summaries (MentalCLOUDS) for summarization tasks across 3 psychotherapy elements: symptom and history, patient discovery, and reflecting.

Model	Variant or parameters	Time (min)	GPU^a^
BART^b^	Base	2.27	A100
T5^c^	Base	18.81	A100
MentalBART	Base	5.94	A100
Flan-T5	Base	16.56	A100
GPT-2	124 million	6.30	A100
GPT-Neo	1.3 billion	32.98	A100
GPT-J	6 billion	44.69	A100
MentalLlama	7	48.27	RTX A6000+RTX A5000
Mistral	7 billion	43.86	RTX A6000+RTX A5000
Phi-2	2.7 billion	9.38	A100

^a^GPU: graphics processing unit.

^b^BART: Bidirectional and Auto-Regressive Transformer.

^c^T5: Text-To-Text Transfer Transformer.

### Ethical Considerations

The study did not involve any human subject research; hence, we did not seek ethics approval.

## Results

We undertook a comprehensive evaluation of the generated session summaries across various architectures, using a dual approach of quantitative and qualitative assessments.

### Quantitative Assessment

#### Overview

This section reports the aspect-based (psychotherapy element–based) summarization results based on the automatic evaluation scores. Given the generative nature of the task, we used standard summarization evaluation metrics such as Recall-Oriented Understudy for Gisting Evaluation (ROUGE)-1, ROUGE-2, ROUGE-L, and BERT Score (BERTScore) along with their corresponding precision, recall, and *F*_1_-score values. As the *F*_1_-score accounts for precision and recall, we compared the performance of the LLMs based on *F*_1_-score values unless stated otherwise. ROUGE [87] assesses the overlap of n-grams (sequences of n consecutive words) between the generated summary and reference summaries. Specifically, this metric measures the number of overlapping units such as n-grams, word sequences, and word pairs in the generated summary evaluated against the gold summary typically created by humans. ROUGE favors the candidate summary with more overlaps with reference summaries. This effectively gives more weight to matching n-grams occurring in multiple reference summaries. This work reports the unigram and bigram ROUGE (namely ROUGE-1 and ROUGE-2) and ROUGE-L evaluations. ROUGE-L takes into account the longest co-occurring n-gram between the candidate and reference summaries. BERTScore [88] is harnessed to gauge the semantic coherence between the generated summaries and their ground truths. Notably, in the context of counseling summaries, which are inherently tied to a domain-specific conversation, we embarked on a meticulous qualitative examination of the generated summaries for individual counseling components.

#### SH Summarization

Table 3 reports the automatic evaluation scores of the LLMs on the summarization task for the SH psychotherapy element. MentalLlama outperformed the other LLMs across all automatic evaluation metrics. For the ROUGE-1 metric, MentalLlama achieved an *F*_1_-score of 30.86, followed by MentalBART with an *F*_1_-score of 28.00. In terms of the ROUGE-2 metric, Mistral was comparable to MentalLlama with a difference of just 0.90 in the *F*_1_-score values. Similarly, for the ROUGE-L metric, Mistral was preceded by MentalLlama by a difference of 2.93 in the *F*_1_-score values.

**Table 3:** Results obtained on Mental Health Counseling-Component–Guided Dialogue Summaries (MentalCLOUDS) for the summarization task on the symptom and history psychotherapy element.

Model	ROUGE^a^-1			ROUGE-2				ROUGE-L			BERTScore^b^		
	Precision	Recall	*F*_1_-score	Precision	Recall	*F*_1_-score	Precision		Recall	*F*_1_-score	Precision	Recall	*F*_1_-score
BART^c^	12.91	28.84	16.26	1.88	5.07	2.47	10.21		23.97	13.19	85.81	85.81	85.81
T5^d^	22.16	19.81	19.74	2.18	1.78	1.85	16.12		14.51	14.36	85.38	85.38	85.38
MentalBART	30.31	29.02	28.00	6.06	5.29	5.46	20.85		20.34	19.40	88.34	88.34	88.34
Flan-T5	21.45	*33.15* ^e^	24.80	3.84	6.08	4.54	17.15		26.53	19.76	86.94	86.94	86.94
GPT-2	6.59	14.62	8.91	1.06	2.34	1.42	5.12		11.37	6.93	83.65	83.65	83.65
GPT-Neo	9.97	19.91	13.01	1.01	2.30	1.38	7.89		15.91	10.33	83.12	83.12	83.12
GPT-J	13.22	29.99	17.88	3.37	*7.96*	4.59	10.71		24.34	14.47	86.28	86.28	86.28
MentalLlama	*33.03*	32.79	*30.86*	*8.66*	6.50	*7.28*	*27.73*		*27.30*	*29.55*	*89.40*	*90.99*	*90.99*
Mistral	29.07	26.56	25.41	7.03	5.20	7.19	25.45		25.61	26.62	83.42	85.96	83.05
Llama-2	28.49	24.17	23.47	6.40	4.68	6.63	22.7		23.04	23.66	82.86	83.80	81.62
Phi-2	21.23	10.42	13.81	1.89	1.43	1.78	14.56		9.19	11.26	84.25	82.00	83.11

^a^ROUGE: Recall-Oriented Understudy for Gisting Evaluation.

^b^BERTScore: Bidirectional Encoder Representations from Transformers Score.

^c^BART: Bidirectional and Auto-Regressive Transformer.

^d^T5: Text-To-Text Transfer Transformer.

^e^The best results are italicized.

#### PD Summarization

The experimental results presented in Table 4 focus on the summarization task for the PD psychotherapy element. Considering the ROUGE-1 metric, MentalLlama demonstrated superior performance compared to the other LLMs. MentalLlama achieved an *F*_1_-score of 30.95, followed by MentalBART (with an *F*_1_-score of 29.94). For the ROUGE-2 metric, GPT-J outperformed the other models, followed by MentalLlama. In addition, in terms of the ROUGE-L metric, the top 2 models with the highest *F*_1_-score values were F1 score models were MentalLlama and Mistral. Finally, MentalBART superseded the other models with an *F*_1_-score of 88.61 with respect to the BERTScore metric. Overall, the scores indicate that LLMs such as MentalLlama and MentalBART, which were pretrained on the mental domain data, show consistent superiority. Notably, the base Mistral model also performed comparably to, and sometimes better than, the models trained on the mental health domain data.

**Table 4:** Results obtained on Mental Health Counseling-Component–Guided Dialogue Summaries (MentalCLOUDS) for the summarization task on the patient discovery psychotherapy element.

Model	ROUGE^a^-1				ROUGE-2				ROUGE-L				BERTScore^b^		
	Precision	Recall	*F*_1_-score	Precision		Recall	*F*_1_-score	Precision		Recall	*F*_1_-score	Precision		Recall	*F*_1_-score
BART^c^	20.82	43.24	26.72	5.97		12.93	7.74	16.38		34.82	21.14	87.35		87.35	87.35
T5^d^	9.43	47.29	15.34	3.03		16.90	5.01	8.39		42.58	13.67	84.77		84.77	84.77
MentalBART	33.51	29.94	29.94	9.36		7.94	8.06	23.39		21.44	21.10	*88.61* ^e^		88.61	*88.61*
Flan-T5	21.08	35.61	24.44	4.81		8.89	5.63	16.13		28.29	18.94	86.52		86.52	86.52
GPT-2	13.66	36.24	19.57	4.08		11.27	5.94	10.93		29.42	15.70	85.21		85.21	85.21
GPT-Neo	12.96	29.93	17.83	2.32		5.44	3.22	9.84		23.10	13.60	82.72		82.72	82.72
GPT-J	19.78	*53.33*	28.85	*12.68*		*35.71*	*18.71*	16.12		*43.33*	23.49	86.43		86.43	86.43
MentalLlama	*24.56*	43.84	*30.95*	9.55		26.01	12.79	*23.77*		38.98	*29.17*	84.63		*88.95*	86.68
Mistral	22.84	39.02	27.54	8.78		25.79	11.35	21.90		35.98	24.02	86.62		87.28	84.49
Llama-2	20.22	34.7	26.1	8.41		21.13	10.39	14.73		21.44	17.79	78.81		88.06	81.48
Phi-2	18.72	9.23	12.45	5.61		4.44	4.96	13.94		8.73	10.98	84.25		82.00	80.05

^a^ROUGE: Recall-Oriented Understudy for Gisting Evaluation.

^b^BERTScore: Bidirectional Encoder Representations from Transformers Score.

^c^BART: Bidirectional and Auto-Regressive Transformer.

^d^T5: Text-To-Text Transfer Transformer.

^e^The best results are italicized.

#### Reflecting

Table 5 reports the automatic evaluation scores on the summarization task for the reflecting psychotherapy element. In terms of the ROUGE-1 metric, MentalLlama and Mistral were the best 2 models, with *F*_1_-score values of 39.52 and 38.33, respectively. Similarly, MentalLlama demonstrated its superiority over the other LLMs in terms of the ROUGE-2, ROUGE-L and BERTScore metrics. Moreover, the scores of the summarization tasks for this psychotherapy element were analogous to those of the previous 2 summarization tasks, namely SH and PD, wherein the mental health–specific LLMs exhibited their superiority over the other LLMs.

**Table 5:** Results obtained on Mental Health Counseling-Component–Guided Dialogue Summaries (MentalCLOUDS) for the summarization task on the reflecting psychotherapy element.

Model	ROUGE^a^-1			ROUGE-2			ROUGE-L			BERTScore^b^		
	Precision	Recall	*F*_1_-score	Precision	Recall	*F*_1_-score	Precision	Recall	*F*_1_-score	Precision	Recall	*F*_1_-score
BART^c^	17.01	23.04	18.08	2.87	4.25	3.22	12.68	17.79	13.66	85.26	85.26	85.26
T5^d^	34.13	19.32	24.31	7.21	3.97	5.04	22.95	12.82	16.21	84.92	84.92	84.92
MentalBART	*34.99* ^e^	36.54	34.46	*10.24*	10.66	10.07	24.52	25.80	24.25	*88.70*	*88.70*	*88.70*
Flan-T5	25.10	41.40	30.15	7.19	12.03	8.64	18.52	31.00	22.36	87.41	87.41	87.41
GPT-2	2.84	7.54	4.08	0.14	0.33	0.20	2.35	6.34	3.39	82.66	82.66	82.66
GPT-Neo	1.14	3.97	1.74	0.00	0.00	0.00	1.14	3.97	1.74	80.88	80.88	80.88
GPT-J	17.60	38.33	23.71	5.07	*13.04*	7.13	14.98	32.85	20.18	86.94	86.94	86.94
MentalLlama	31.68	*54.76*	*39.52*	8.26	11.99	*10.17*	*27.13*	*37.59*	*26.56*	84.77	86.92	87.43
Mistral	29.15	49.28	38.33	8.42	11.87	8.34	24.41	34.20	23.44	78.83	79.97	84.81
Llama-2	26.93	43.81	31.22	6.10	9.23	8.24	16.82	20.67	16.21	78.93	86.05	82.19
Phi-2	10.61	5.21	6.91	0.94	0.71	0.89	7.28	4.60	5.53	86.94	82.17	84.49

^a^ROUGE: Recall-Oriented Understudy for Gisting Evaluation.

^b^BERTScore: Bidirectional Encoder Representations from Transformers Score.

^c^BART: Bidirectional and Auto-Regressive Transformer.

^d^T5: Text-To-Text Transfer Transformer.

^e^The best results are italicized.

### Qualitative Assessment by Experts

#### Expert Panel Composition and Evaluation Framework

To conduct a comprehensive expert assessment, 5 health care professionals were employed to assess the clinical appropriateness of the summaries produced by the LLMs based on the evaluation framework postulated by Sekhon et al [69]. Of the 5 health care professionals, 2 (40%) were clinical psychologists and 3 (60%) were psychiatrists and medical practitioners; 4 (80%) were male and 1 (20%) was female; and their ages ranged from 40 to 55 years. Furthermore, each health care professional possessed more than a decade of therapeutic experience.

The evaluation framework encompasses 6 crucial parameters: affective attitude, burden, ethicality, coherence, opportunity costs, and perceived effectiveness. The experts evaluated each session summary against these acceptability parameters, assigning continuous ratings on a scale ranging from 0 to 2, where a higher rating signified enhanced acceptability. In addition, we incorporated a new parameter: the extent of hallucination. It is categorical: 0=extensive hallucination observed, 1=*minimal hallucination observed*, and 2=*no hallucination observed*. These evaluative dimensions are defined in Table 6.

Table 7 reports the clinical experts’ scores averaged over their ratings. The clinical acceptability framework [69] involves 6 parameters: affective attitude, burden, ethicality, coherence, opportunity costs, and perceived effectiveness (refer to Table 6 for more details). We selected the 3 best LLMs (MentalLlama, Mistral, and MentalBART) for the expert evaluation based on the automatic evaluation results. Notably, Mistral outperformed the other 2 LLMs across all metrics, although the other 2 LLMs were fine-tuned on mental health domain data. Overall, all raters were more aligned in rating the MentalBART model with less variance than the other 2 LLMs across all metrics. However, all 3 LLMs were rated higher on the surface-level–characteristic metric (burden) or subjective metric (affective attitude) than the opportunity costs and efficacy metrics (perceived effectiveness). The poor scores of all 3 models on the more sensitive aspects, that is, the overall efficacy and the opportunity costs, indicate that these models share the same weakness and are not suitable for clinical use as they stand now.

**Table 6:** Explanation of the experts’ evaluation metrics based on the evaluation framework postulated by Sekhon et al [69].

Construct	Definition	Application
Affective attitude	How an individual feels about an intervention	What are your perceptions of the summarization based upon your clinical knowledge?
Burden	Perceived amount of effort required to participate	How much effort is required to understand the summarization (consider spelling, grammar, and overall interpretation)?
Ethicality	Extent to which this is a good fit with your organization’s value system	How does this align with your respective code of ethics? Are there concerns?
Coherence	Extent to which the intervention is understood	How well the summaries are understood
Opportunity costs	The extent to which one would benefit from using this intervention	Pros and cons of using this intervention in your respective setting
Perceived effectiveness	Extent to which this intervention will perform in the intended setting	How well this will perform in your clinical setting
Extent of hallucination	Extent to which this intervention is hallucinated	The generated text is incorrect, nonsensical, or contains global information apart from the context of the conversation

**Table 7:** Qualitative evaluation by human experts, with scores averaged from the 5 expert raters. The variances among the raters’ scores are also shown.

Model		Affective attitude	Burden	Ethicality	Intervention coherence	Opportunity costs	Perceived effectiveness
**Mistral**							
	Values, mean (SD)	*1.12 (0.47)* ^a^	*1.33 (0.32)*	*1.42 (0.37)*	*1.13 (0.45)*	*0.98 (0.47)*	*0.90 (0.51)*
	Variance	0.22	0.10	0.14	0.20	0.22	0.26
**MentalLlama**							
	Values, mean (SD)	*1.12 (*0.37*)*	*1.33 (*0.22*)*	1.36 (0.32)	1.06 (0.36)	0.94 (0.39)	0.88 (0.45)
	Variance	0.14	0.05	0.10	0.13	0.15	0.20
**MentalBART**							
	Values, mean (SD)	0.95 (0.28)	1.28 (0.14)	1.33 (0.36)	1.01 (0.22)	0.84 (0.33)	0.76 (0.4)
	Variance	0.08	0.02	0.13	0.05	0.11	0.16

^a^The best results are italicized.

#### Extent of Hallucination

The evaluation of hallucination identification in a set of 39 conversations was divided into 3 hallucination levels: *no hallucination* *observed*, minimal hallucination observed, and extensive hallucination observed. These categories essentially determine how well the response is consistent with the context and whether it is also incorrect, nonsensical, or contains global information beyond the scope of the conversation. The results are summarized in Table 8. The data show fluctuations in how the phenomenon of hallucination is perceived among different models and stress the importance of reviewing evaluations from numerous appraisers for a complete assessment. Here, we report the average hallucination-level frequencies rated by the 5 evaluators. Subsequently, we provide the percentage of the hallucination-level frequency against the total 39 instances. Of the test conversations, the majority of cases (n=39, 76%), on average, demonstrated *no hallucination observed*: Mistral and MentalBART achieved rates of 75% and 76%, respectively, while MentalLlama showed a slightly higher value: 77%. Among the samples where minimal hallucination observed was reported, all 3 models fell within a similar range: Mistral and MentalLlama had rates of 13% and 14%, respectively, while MentalBART showed a slightly elevated value of 18%. Notably, the models exhibited lower rates in terms of the extensive hallucination observed category, with Mistral at only 11%, MentalLlama at 7%, and MentalBART at 5%. These data confirm the capability of these AI models to faithfully follow whenever there is no hallucination and underscore their ability to detect more subtle degrees of hallucination across the various tasks on which they were tested.

The results are consistently adequate across all 3 models, with a relatively equal distribution of the level of hallucination observed by different raters. Importantly, all 3 models exhibited a significant number of cases with *no hallucination observed*, indicating reliable performance and implying their ability to maintain fidelity to the original content.

**Table 8:** Hallucination-level frequency marked by experts for the top 3 large language models. The average of hallucination-level frequencies for each rater is reported.

Hallucination level	Mistral (%), mean (SD)	MentalLlama (%), mean (SD)	MentalBART (%), mean (SD)
No hallucination observed	29.3 (1.64)	30.3 (2.03)	29.7 (1.58)
Minimal hallucination observed	5.1 (0.51)	5.6 (1.07)	7.3 (0.96)
Extensive hallucination observed	4.3 (1.34)	3 (1)	2 (0.67)

## Discussion

### Principal Findings

In this study, we assessed 11 state-of-the-art LLMs on the aspect-based summarization task of mental health therapy conversations. These therapy conversations are long, and it requires a good amount of effort to gain insights from reading them. To address this, we summarized these long conversations, thereby reducing the efforts of the experts. We further proposed MentalCLOUDS, which provides aspect-based summaries of each conversation.

Specifically, we benchmarked the 11 LLMs for aspect-based summarization and evaluated them using both automatic and human evaluation approaches. The automatic evaluation scores revealed the superiority of the LLMs trained on mental health domain data. Two domain-specific LLMs, MentalLlama and MentalBART, consistently outperformed the rest of the LLMs across all aspects. Notably, although Mistral is not specifically trained on mental health domain data, its scores are comparable to those of MentalLlama, the overall best-performing model.

This work also showcased the prowess of decoder-only LLMs compared to strong encoder-decoder–based LLMs. Typically, encoder-decoder models favor sequence-to-sequence tasks such as summarization, where a sequence of input texts is mapped to a sequence of output texts. However, the decoder-based models, that is, MentalLlama and Mistral, consistently outperformed the encoder-decoder models such as BART, T5, and Flan-T5. The only exception was MentalBART because it is fine-tuned on the mental health data set.

The counseling data set was curated from multiple multimedia web-based sources such as YouTube transcripts [47]. Hence, most of these natural conversations are incoherent and grammatically unfluent. Even with these imperfections, the LLMs were mostly able to construct meaningful summaries that contained coherent narratives with a clear beginning and end. However, the models did not do as well with the structure separation of the information. The SH, PD, and reflection sections frequently overlapped, posing clinical and legal problems. History is considered clinically sacrosanct and should not be contaminated by the therapist’s interpretation, and it is also citable in legal cases as client evidence, while interpretations are not. The models were also unable to identify psychotherapy types (eg, cognitive behavioral therapy) and therapy techniques, which form an integral part of counseling notes; for example, when participants are engaged in using a motivational interviewing framework, the essential processes and their outcomes, which a human summarizer would have recorded, failed to find a place in the LLM summaries. Important negative histories gathered during the session, such as the history of suicide risk or substance use, were also not recorded; and in at least 1 instance, the presence of suicide risk was not identified. In general, the models exhibited stronger performance in handling medical histories and examinations but struggled when faced with more technical and sensitive aspects, such as conversations related to actual therapeutic strategies.

### Limitations

It is crucial to address the limitations of this study for a comprehensive understanding. First, this work aimed to benchmark the efficacy of only 11 LLMs on the aspect-based summarization task. Second, for faster and easier reproduction of the results, we did not assess models larger than 7 billion parameters; however, such models can be part of future examinations. Third, for the initial study and to promote research in this field, only open-source models were assessed in this work. However, inspecting closed models such as ChatGPT, Claude, and Gemini can be an interesting future research avenue. Finally, this work explored only 3 aspects (counseling components) of the conversation. However, conversations are subjective and can have >3 components. In addition, the counseling sessions in this work represented a certain demographic region (American) and thus may not apply to therapy counseling for other demographics.

### Conclusions

Our study benchmarked the efficacy and role of LLMs in counseling-component–guided summarization tasks. In doing so, we introduced a new data set, MentalCLOUDS, which comprises summaries corresponding to 3 counseling components. The experimental results confirmed the superiority of the LLMs fine-tuned on mental health domain data (MentalLlama and MentalBART) over the out-of-the-box LLMs. Notably, the out-of-the-box Mistral model seemed comparable to, and sometimes better than, the LLMs fine-tuned on mental health domain data. However, as per the experts’ evaluation, these LLMs often failed to distinguish between the counseling components during summary generation. Overall, these models excelled in managing medical histories and examinations but faced challenges with technical and sensitive aspects, such as therapy conversations, thereby limiting their clinical utility as they stand now.

## Abbreviations

AI: artificial intelligence
BART: Bidirectional and Auto-Regressive Transformer
BERT: Bidirectional Encoder Representations from Transformers
BERTScore: Bidirectional Encoder Representations from Transformers Score
LLM: large language model
MEMO: Mental Health Summarization
MentalCLOUDS: Mental Health Counseling-Component–Guided Dialogue Summaries
PD: patient discovery
PICO: Population, Intervention, Comparison, and Outcomes
ROUGE: Recall-Oriented Understudy for Gisting Evaluation
SH: symptom and history
SLM: small language model
T5: Text-to-Text Transfer Transformer

## Data Availability

The data sets generated and analyzed during this study are available from the corresponding author on reasonable request.

## References

[1] Chen P, Verma R (2006). A query-based medical information summarization system using ontology knowledge. Proceedings of the 19th IEEE Symposium on Computer-Based Medical Systems.

[2] Chen YC, Bansal M (2018). Fast abstractive summarization with reinforce-selected sentence rewriting. Proceedings of the 56th Annual Meeting of the Association for Computational Linguistics.

[3] Narayan S, Cohen SB, Lapata M (2018). Don’t give me the details, just the summary! Topic-aware convolutional neural networks for extreme summarization. Proceedings of the 2018 Conference on Empirical Methods in Natural Language Processing.

[4] Moratanch N, Chitrakala S (2017). A survey on extractive text summarization. Proceedings of the International Conference on Computer, Communication and Signal Processing.

[5] Gupta S, Gupta SK (2019). Abstractive summarization: an overview of the state of the art. Expert Syst Appl.

[6] Tuggener D, Mieskes M, Deriu J, Cieliebak M (2021). Are we summarizing the right way? A survey of dialogue summarization data sets. Proceedings of the Third Workshop on New Frontiers in Summarization.

[7] Konovalov S, Scotch M, Post L, Brandt C (2010). Biomedical informatics techniques for processing and analyzing web blogs of military service members. J Med Internet Res.

[8] Strauss J, Peguero AM, Hirst G (2013). Machine learning methods for clinical forms analysis in mental health. Stud Health Technol Inform.

[9] Kennedy SH, Lam RW, McIntyre RS, Tourjman SV, Bhat V, Blier P, Hasnain M, Jollant F, Levitt AJ, MacQueen GM, McInerney SJ, McIntosh D, Milev RV, Müller DJ, Parikh SV, Pearson NL, Ravindran AV, Uher R (2016). Canadian network for mood and anxiety treatments (CANMAT) 2016 clinical guidelines for the management of adults with major depressive disorder: section 3. Pharmacological treatments. Can J Psychiatry.

[10] Tran T, Kavuluru R (2017). Predicting mental conditions based on "history of present illness" in psychiatric notes with deep neural networks. J Biomed Inform.

[11] Chen YP, Chen YY, Lin JJ, Huang CH, Lai F (2020). Modified bidirectional encoder representations from transformers extractive summarization model for hospital information systems based on character-level tokens (AlphaBERT): development and performance evaluation. JMIR Med Inform.

[12] Devlin J, Chang MW, Lee K, Toutanova K (2019). BERT: pre-training of deep bidirectional transformers for language understanding. Proceedings of the 2019 Conference of the North American Chapter of the Association for Computational Linguistics: Human Language Technologies.

[13] Ive J, Viani N, Kam J, Yin L, Verma S, Puntis S, Cardinal RN, Roberts A, Stewart R, Velupillai S (2020). Generation and evaluation of artificial mental health records for Natural Language Processing. NPJ Digit Med.

[14] Afzal M, Alam F, Malik KM, Malik GM (2020). Clinical context-aware biomedical text summarization using deep neural network: model development and validation. J Med Internet Res.

[15] Manas G, Aribandi V, Kursuncu U, Alambo A, Shalin VL, Thirunarayan K, Beich J, Narasimhan M, Sheth A (2021). Knowledge-infused abstractive summarization of clinical diagnostic interviews: framework development study. JMIR Ment Health.

[16] Zhang L, Negrinho R, Ghosh A, Jagannathan V, Hassanzadeh HR, Schaaf T, Gormley MR (2021). Leveraging pretrained models for automatic summarization of doctor-patient conversations. Proceedings of the 2021 Conference on Empirical Methods in Natural Language Processing.

[17] Zafari H, Zulkernine F (2021). Chatsum: an intelligent medical chat summarization tool. Proceedings of the 2021 IEEE EMBS International Conference on Biomedical and Health Informatics.

[18] Nallapati R, Zhou B, Gu̇lçehre Ç, dos Santos C, Xiang B (2016). Abstractive text summarization using sequence-to-sequence RNNs and beyond. Proceedings of the 20th SIGNLL Conference on Computational Natural Language Learning.

[19] Vaswani A, Shazeer N, Parmar N, Uszkoreit J, Jones L, Gomez AN, Kaiser L, Polosukhin I Attention is all you need. arXiv.

[20] See A, Liu PJ, Manning CD Get to the point: summarization with pointer-generator networks. arXiv.

[21] Blei DM, Ng AY, Jordan MI (2003). Latent dirichlet allocation. J Mach Learn Res.

[22] Song Y, Tian Y, Wang N, Xia F (2020). Summarizing medical conversations via identifying important utterances. Proceedings of the 28th International Conference on Computational Linguistics.

[23] Quiroz JC, Laranjo L, Kocaballi AB, Briatore A, Berkovsky S, Rezazadegan D, Coiera E (2020). Identifying relevant information in medical conversations to summarize a clinician-patient encounter. Health Informatics J.

[24] Krishna K, Khosla S, Bigham JP, Lipton ZC Generating SOAP notes from doctor-patient conversations using modular summarization techniques. arXiv..

[25] Titov I, McDonald R (2008). A joint model of text and aspect ratings for sentiment summarization. Proceedings of the 46th Annual Meeting of the Association for Computational Linguistics: Human Language Technologies.

[26] Lu Y, Zhai C, Sundaresan N (2009). Rated aspect summarization of short comments. Proceedings of the 18th International Conference on World Wide Web.

[27] Yang M, Qu Q, Shen Y, Liu Q, Zhao W, Zhu J (2018). Aspect and sentiment aware abstractive review summarization. Proceedings of the 27th International Conference on Computational Linguistics.

[28] Wang L, Ling W Neural network-based abstract generation for opinions and arguments. arXiv..

[29] Frermann L, Klementiev A (2019). Inducing document structure for aspect-based summarization. Proceedings of the 57th Annual Meeting of the Association for Computational Linguistics.

[30] Krishna K, Srinivasan BV (2018). Generating topic-oriented summaries using neural attention. Proceedings of the 2018 Conference of the North American Chapter of the Association for Computational Linguistics: Human Language Technologies.

[31] Hayashi H, Budania P, Wang P, Ackerson C, Neervannan R, Neubig G WikiAsp: a dataset for multi-domain aspect-based summarization. arXiv..

[32] Yang X, Song K, Cho S, Wang X, Pan X, Petzold L, Yu D OASum: large-scale open domain aspect-based summarization. arXiv..

[33] Beltagy I, Peters ME, Cohan A Longformer: the long-document transformer. arXiv..

[34] Joshi A, Katariya N, Amatriain X, Kannan A Dr. Summarize: global summarization of medical dialogue by exploiting local structures. arXiv..

[35] Liu Z, Ng A, Lee S, Aw AT, Chen NF Topic-aware pointer-generator networks for summarizing spoken conversations. arXiv..

[36] Kazi N, Kahanda I (2019). Automatically generating psychiatric case notes from digital transcripts of doctor-patient conversations. Proceedings of the 2nd Clinical Natural Language Processing Workshop.

[37] Gundogdu B, Pamuksuz U, Chung JH, Telleria JM, Liu P, Khan F, Chang PJ (2023). Customized impression prediction from radiology reports using BERT and LSTMs. IEEE Trans Artif Intell.

[38] Han Q, Yang Z, Lin H, Qin T (2024). Let topic flow: a unified topic-guided segment-wise dialogue summarization framework. IEEE/ACM Trans Audio Speech Lang Process.

[39] Zhang M, You D, Wang S (2024). Novel framework for dialogue summarization based on factual-statement fusion and dialogue segmentation. PLoS One.

[40] Chintagunta B, Katariya N, Amatriain X, Kannan A Medically aware GPT-3 as a data generator for medical dialogue summarization. arXiv..

[41] Brown TB, Mann B, Ryder N, Subbiah M, Kaplan J, Dhariwal P, Neelakantan A, Shyam P, Sastry G, Askell A, Agarwal S, Herbert-Voss A, Krueger G, Henighan T, Child R, Ramesh A, Ziegler DM, Wu J, Winter C, Hesse C, Chen M, Sigler E, Litwin M, Gray S, Chess B, Clark J, Berner C, McCandlish S, Radford A, Sutskever I, Amodei D Language models are few-shot learners. arXiv..

[42] Liu Y, Ju S, Wang J (2024). Exploring the potential of ChatGPT in medical dialogue summarization: a study on consistency with human preferences. BMC Med Inform Decis Mak.

[43] Lewis M, Liu Y, Goyal N, Ghazvininejad M, Mohamed A, Levy O, Stoyanov V, Zettlemoyer L BART: denoising sequence-to-sequence pre-training for natural language generation, translation, and comprehension. arXiv..

[44] Liu Y Fine-tune BERT for extractive summarization. arXiv..

[45] Van Veen D, Van Uden C, Blankemeier L, Delbrouck JB, Aali A, Bluethgen C, Pareek A, Polacin M, Reis EP, Seehofnerová A, Rohatgi N, Hosamani P, Collins W, Ahuja N, Langlotz CP, Hom J, Gatidis S, Pauly J, Chaudhari AS (2024). Adapted large language models can outperform medical experts in clinical text summarization. Nat Med.

[46] Singh LG, Mao J, Mutalik R, Middleton SE (2024). Extracting and summarizing evidence of suicidal ideation in social media contents using large language models. Proceedings of the 9th Workshop on Computational Linguistics and Clinical Psychology.

[47] Srivastava A, Suresh T, Lord SP, Akhtar MS, Chakraborty T (2022). Counseling summarization using mental health knowledge guided utterance filtering. Proceedings of the 28th ACM SIGKDD Conference on Knowledge Discovery and Data Mining.

[48] Raffel C, Shazeer N, Roberts A, Lee K, Narang S, Matena M, Zhou Y, Li W, Liu PJ Exploring the limits of transfer learning with a unified text-to-text transformer. arXiv..

[49] Shah DJ, Yu L, Lei T, Barzilay R Nutri-bullets: summarizing health studies by composing segments. arXiv..

[50] Park JW Continual BERT: continual learning for adaptive extractive summarization of COVID-19 literature. arXiv..

[51] Shah D, Yu L, Lei T, Barzilay R (2021). Nutri-bullets hybrid: consensual multi-document summarization. Proceedings of the 2021 Conference of the North American Chapter of the Association for Computational Linguistics: Human Language Technologies.

[52] Wallace BC, Saha S, Soboczenski F, Marshall IJ (2021). Generating (factual?) narrative summaries of RCTs: experiments with neural multi-document summarization. AMIA Jt Summits Transl Sci Proc.

[53] Zhu Y, Yang X, Wu Y, Zhang W (2023). Leveraging summary guidance on medical report summarization. IEEE J Biomed Health Inform.

[54] Jiang Z, Cai X, Yang L, Gao D, Zhao W, Han J, Liu J, Shen D, Liu T (2023). Learning to summarize Chinese radiology findings with a pre-trained encoder. IEEE Trans Biomed Eng.

[55] Van Veen D, Van Uden C, Attias M, Pareek A, Bluethgen C, Polacin M, Chiu W, Delbrouck JB, Chaves JM, Langlotz CP, Chaudhari AS, Pauly J RadAdapt: radiology report summarization via lightweight domain adaptation of large language models. arXiv..

[56] Jo HS, Park K, Jung SM (2019). A scoping review of consumer needs for cancer information. Patient Educ Couns.

[57] Finney Rutten LJ, Blake KD, Greenberg-Worisek AJ, Allen SV, Moser RP, Hesse BW (2019). Online health information seeking among US adults: measuring progress toward a healthy people 2020 objective. Public Health Rep.

[58] Mrini K, Dernoncourt F, Yoon S, Bui T, Chang W, Farcas E, Nakashole N (2021). A gradually soft multi-task and data-augmented approach to medical question understanding. Proceedings of the 59th Annual Meeting of the Association for Computational Linguistics and the 11th International Joint Conference on Natural Language Processing.

[59] Roberts K, Demner-Fushman D (2016). Interactive use of online health resources: a comparison of consumer and professional questions. J Am Med Inform Assoc.

[60] Kolhatkar G, Paranjape A, Gokhale O, Kadam D (2023). Team converge at ProbSum 2023: abstractive text summarization of patient progress notes. Proceedings of the 22nd Workshop on Biomedical Natural Language Processing and BioNLP Shared Tasks.

[61] Liu M, Zhang D, Tan W, Zhang H (2023). DeakinNLP at ProbSum 2023: clinical progress note summarization with rules and language models. Proceedings of the 22nd Workshop on Biomedical Natural Language Processing and BioNLP Shared Tasks.

[62] Gao Y, Dligach D, Miller T, Churpek MM, Afshar M Overview of the problem list summarization (ProbSum) 2023 shared task on summarizing patients' active diagnoses and problems from electronic health record progress notes. arXiv..

[63] Gao Y, Miller T, Xu D, Dligach D, Churpek MM, Afshar M (2022). Summarizing patients' problems from hospital progress notes using pre-trained sequence-to-sequence models. Proc Int Conf Comput Ling.

[64] Shing HC, Shivade C, Pourdamghani N, Nan F, Resnik P, Oard D, Bhatia P Towards clinical encounter summarization: learning to compose discharge summaries from prior notes. arXiv..

[65] Ando K, Okumura T, Komachi M, Horiguchi H, Matsumoto Y (2022). Exploring optimal granularity for extractive summarization of unstructured health records: analysis of the largest multi-institutional archive of health records in Japan. PLOS Digit Health.

[66] Zhao B, Zan H, Niu C, Chang H, Zhang K (2023). Automatic generation of discharge summary of EMRs based on multi-granularity information fusion. Proceedings of the 9th China Health Information Processing Conference.

[67] Jain R, Saha T, Lalwani J, Saha S (2023). Can you summarize my learnings? Towards perspective-based educational dialogue summarization. Proceedings of the 2023 Conference on Empirical Methods in Natural Language Processing.

[68] Saha T, Reddy S, Das A, Saha S, Bhattacharyya P (2022). A shoulder to cry on: towards a motivational virtual assistant for assuaging mental agony. Proceedings of the 2022 Conference of the North American Chapter of the Association for Computational Linguistics: Human Language Technologies.

[69] Sekhon M, Cartwright M, Francis JJ (2017). Acceptability of healthcare interventions: an overview of reviews and development of a theoretical framework. BMC Health Serv Res.

[70] Poria S, Majumder N, Mihalcea R, Hovy E Emotion recognition in conversation: research challenges, datasets, and recent advances. arXiv..

[71] Chen H, Liu X, Yin D, Tang J (2017). A survey on dialogue systems: recent advances and new frontiers. ACM SIGKDD Explor Newsl.

[72] Radford A, Wu J, Child R, Luan D, Amodei D, Sutskever I (2019). Language models are unsupervised multitask learners. OpenAI Blog.

[73] Black S, Biderman S, Hallahan E, Anthony Q, Gao L, Golding L, He H, Leahy C, McDonell K, Phang J, Pieler M, Prashanth US, Purohit S, Reynolds L, Tow J, Wang B, Weinbach S GPT-NeoX-20B: an open-source autoregressive language model. arXiv..

[74] Gao L, Biderman S, Black S, Golding L, Hoppe T, Foster C, Phang J, He H, Thite A, Nabeshima N, Presser S, Leahy C The pile: an 800GB dataset of diverse text for language modeling. arXiv..

[75] Li R, Su J, Duan C, Zheng S Linear attention mechanism: an efficient attention for semantic segmentation. arXiv..

[76] Shazeer N, Mirhoseini A, Maziarz K, Davis A, Le Q, Hinton G, Dean J Outrageously large neural networks: the sparsely-gated mixture-of-experts layer. arXiv..

[77] Ho J, Kalchbrenner N, Weissenborn D, Salimans T Axial attention in multidimensional transformers. arXiv..

[78] Wang B (2021). Mesh-transformer-JAX: model-parallel implementation of transformer language model with JAX. GitHub.

[79] Chung HW, Hou L, Longpre S, Zoph B, Tay Y, Fedus W, Li Y, Wang X, Dehghani M, Brahma S, Webson A, Gu SS, Dai Z, Suzgun M, Chen X, Chowdhery A, Castro-Ros A, Pellat M, Robinson K, Valter D, Narang S, Mishra G, Yu A, Zhao V, Huang Y, Dai A, Yu H, Petrov S, Chi EH, Dean J, Devlin J, Roberts A, Zhou D, Le QV, Wei J Scaling instruction-finetuned language models. arXiv..

[80] Jiang AQ, Sablayrolles A, Mensch A, Bamford C, Chaplot DS, de las Casas D, Bressand F, Lengyel G, Lample G, Saulnier L, Lavaud LR, Lachaux MA, Stock P, Scao TL, Lavril T, Wang T, Lacroix T, Sayed WE Mistral 7B. arXiv..

[81] Ainslie J, Lee-Thorp J, de Jong, M, Zemlyanskiy Y, Lebrón F, Sanghai S GQA: training generalized multi-query transformer models from multi-head checkpoints. arXiv..

[82] Yang K, Zhang T, Kuang Z, Xie Q, Ananiadou S, Huang J MentaLLaMA: interpretable mental health analysis on social media with large language models. arXiv..

[83] Touvron H, Martin L, Stone K, Albert P, Almahairi A, Babaei Y, Bashlykov N, Batra S, Bhargava P, Bhosale S, Bikel D, Blecher L, Ferrer CC, Chen M, Cucurull G, Esiobu D, Fernandes J, Fu J, Fu W, Fuller B, Gao C, Goswami V, Goyal N, Hartshorn A, Hosseini S, Hou R, Inan H, Kardas M, Kerkez V, Khabsa M, Kloumann I, Korenev A, Koura PS, Lachaux MA, Lavril T, Lee J, Liskovich D, Lu Y, Mao Y, Martinet X, Mihaylov T, Mishra P, Molybog I, Nie Y, Poulton A, Reizenstein J, Rungta R, Saladi K, Schelten A, Silva R, Smith EM, Subramanian R, Tan XE, Tang B, Taylor R, Williams A, Kuan J, Xu P, Yan Z, Zarov I, Zhang Y, Fan A, Kambadur M, Narang S, Rodriguez A, Stojnic R, Edunov S, Scialom T Llama 2: open foundation and fine-tuned chat models. arXiv..

[84] Ouyang L, Wu J, Jiang X, Almeida D, Wainwright CL, Mishkin P, Zhang C, Agarwal S, Slama K, Ray A, Schulman J, Hilton J, Kelton F, Miller L, Simens M, Askell A, Welinder P, Christiano P, Leike J, Lowe R Training language models to follow instructions with human feedback. arXiv..

[85] Christiano P, Leike J, Brown TB, Martic M, Legg S, Amodei D Deep reinforcement learning from human preferences. arXiv..

[86] Gunasekar S, Zhang Y, Aneja J, Mendes CC, Del Giorno A, Gopi S, Javaheripi M, Kauffmann P, de Rosa G, Saarikivi O, Salim A, Shah S, Behl HS, Wang X, Bubeck S, Eldan R, Kalai AT, Lee YT, Li Y Textbooks are all you need. arXiv..

[87] Lin CY (2004). ROUGE: a package for automatic evaluation of summaries. Proceedings of the Workshop on Text Summarization Branches Out (WAS 2004).

[88] Zhang T, Kishore V, Wu F, Weinberger KQ, Artzi Y (2020). BERTScore: evaluating text generation with BERT. International Conference on Learning Representations.

